# Seizures and Hyperleukocytosis Revealing New-Onset Acute Myeloid Leukemia During Acute COVID-19 Infection

**DOI:** 10.7759/cureus.106799

**Published:** 2026-04-10

**Authors:** Vismay Patel, Rubba S Khan, Huma Irfan, Mohammed Mirza, Sujanty Rajaram, Rehan Shah

**Affiliations:** 1 Internal Medicine, Hudson Regional Health (HRH) Bayonne University Hospital, Bayonne, USA; 2 Critical Care Medicine, Hudson Regional Health (HRH) Bayonne University Hospital, Bayonne, USA; 3 Rheumatology, Hudson Regional Health (HRH) Bayonne University Hospital, Bayonne, USA

**Keywords:** acute myeloid leukemia (aml), covid-19, infection, leukostasis, seizures

## Abstract

Acute myeloid leukemia (AML) with hyperleukocytosis is a hematologic emergency associated with high early mortality. Viral infections, including SARS-CoV-2, have been proposed as potential factors that may unmask previously undiagnosed leukemia or accelerate disease progression in predisposed individuals. We report the case of a previously healthy 61-year-old man who presented after recent travel with progressive fatigue, dizziness, fever, and multiple generalized seizures. Initial evaluation revealed COVID-19 infection with profound cytopenias and hyperleukocytosis, including a white blood cell (WBC) count of 132.9 × 10⁹/L, hemoglobin of 5.4 g/dL, and platelets of 11 × 10⁹/L. Peripheral smear demonstrated 36%-40% circulating myeloblasts with Auer rods, consistent with AML. Neuroimaging showed no intracranial hemorrhage. Laboratory evaluation revealed hyperuricemia, markedly elevated lactate dehydrogenase, hyperlactatemia (6.5 mmol/L), and acute kidney injury, concerning for early tumor lysis physiology.

The patient was admitted to the intensive care unit and treated with hydroxyurea for cytoreduction, tumor lysis prophylaxis with allopurinol, transfusion support, and COVID-19-directed therapy. His leukocyte count decreased from 132.9 × 10⁹/L to 32.5 × 10⁹/L within 72 hours, with clinical stabilization. After initial cytoreduction and metabolic improvement, he was transferred to a tertiary oncology center for definitive induction therapy.

This case raises the possibility that COVID-19-associated immune dysregulation may contribute to the clinical presentation of previously undiagnosed leukemia and underscores the importance of rapid cytoreduction, tumor lysis prevention, and multidisciplinary management in patients presenting with hyperleukocytic AML.

## Introduction

Acute myeloid leukemia (AML) is a heterogeneous group of hematopoietic malignancies characterized by clonal proliferation of myeloid precursors leading to bone marrow failure. It most commonly affects older adults and is frequently associated with antecedent hematologic disorders or prior cytotoxic exposure, although de novo cases in previously healthy individuals are well described [1].

Management strategies for hyperleukocytosis, including cytoreduction and leukapheresis, have been studied extensively in patients with AML [2].

Hyperleukocytosis, generally defined as a white blood cell (WBC) count greater than 100 × 10⁹/L, occurs in approximately 10%-20% of AML cases and is associated with high early mortality due to leukostasis, disseminated intravascular coagulation, and tumor lysis syndrome [1,3]. Leukostasis results from increased blood viscosity and microvascular obstruction, leading to impaired tissue perfusion, most commonly affecting the lungs and central nervous system. Neurologic manifestations such as seizures are uncommon but represent severe disease.

SARS-CoV-2 infection has been linked to immune dysregulation and cytokine-mediated myeloid activation. Emerging reports suggest that COVID-19 infection may unmask or accelerate acute leukemia in susceptible individuals through inflammatory and proliferative signaling pathways [4,5].

## Case presentation

A previously healthy 61-year-old man presented to the emergency department after returning from a cruise with progressive fatigue, dizziness, and fever. He reported approximately two weeks of worsening weakness, poor appetite, and intermittent chills. On the day of presentation, he experienced six generalized tonic-clonic seizures associated with head trauma.

On arrival, he appeared pale and lethargic. Vital signs included a temperature of 99.9°F, heart rate of 158 beats per minute, blood pressure of 102/68 mmHg, respiratory rate of 20 breaths per minute, and oxygen saturation of 95% on nasal cannula.

**Figure 1:**
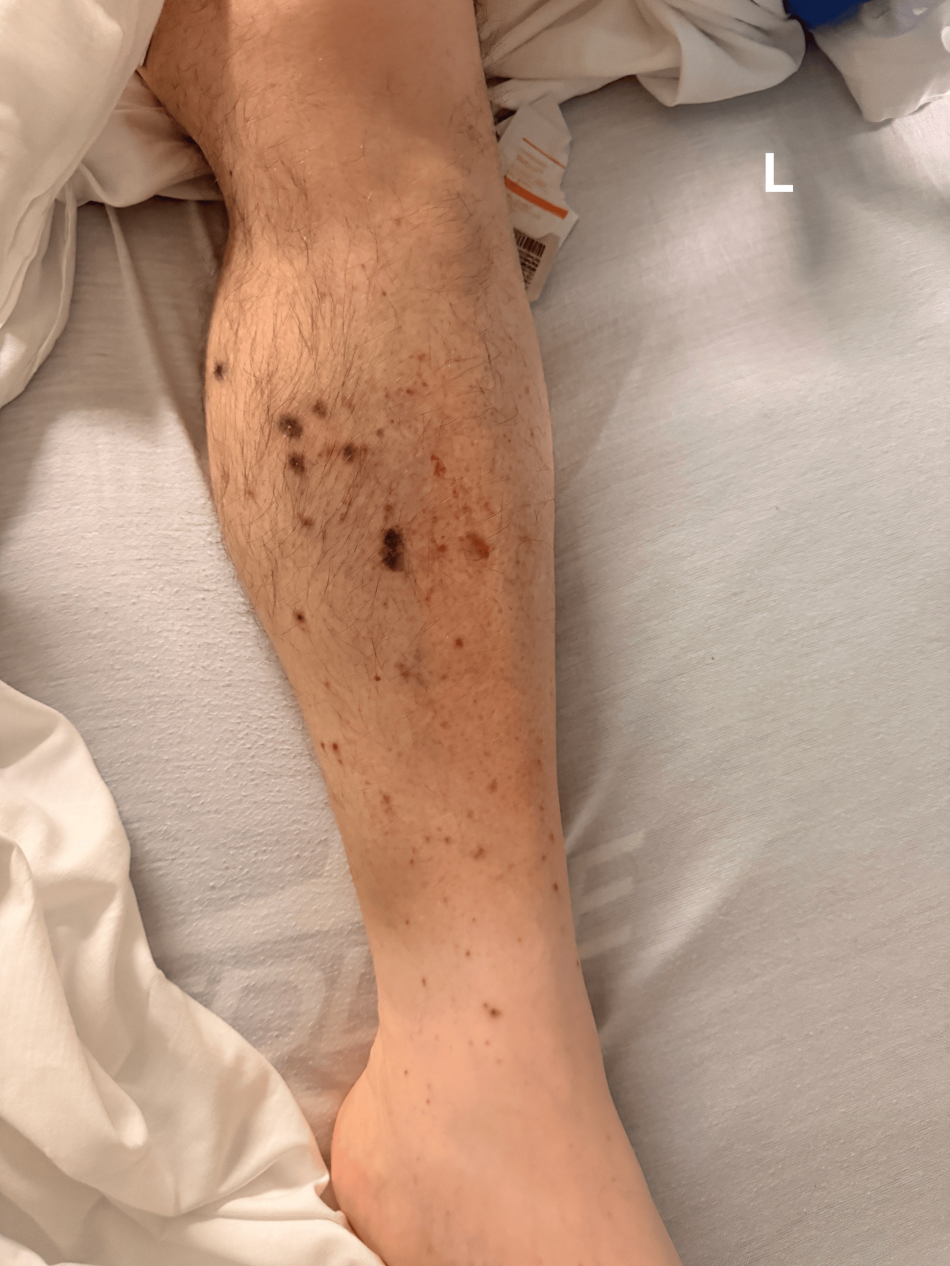
Physical examination revealed a maculopapular rash over the left lower extremity.

**Figure 2:**
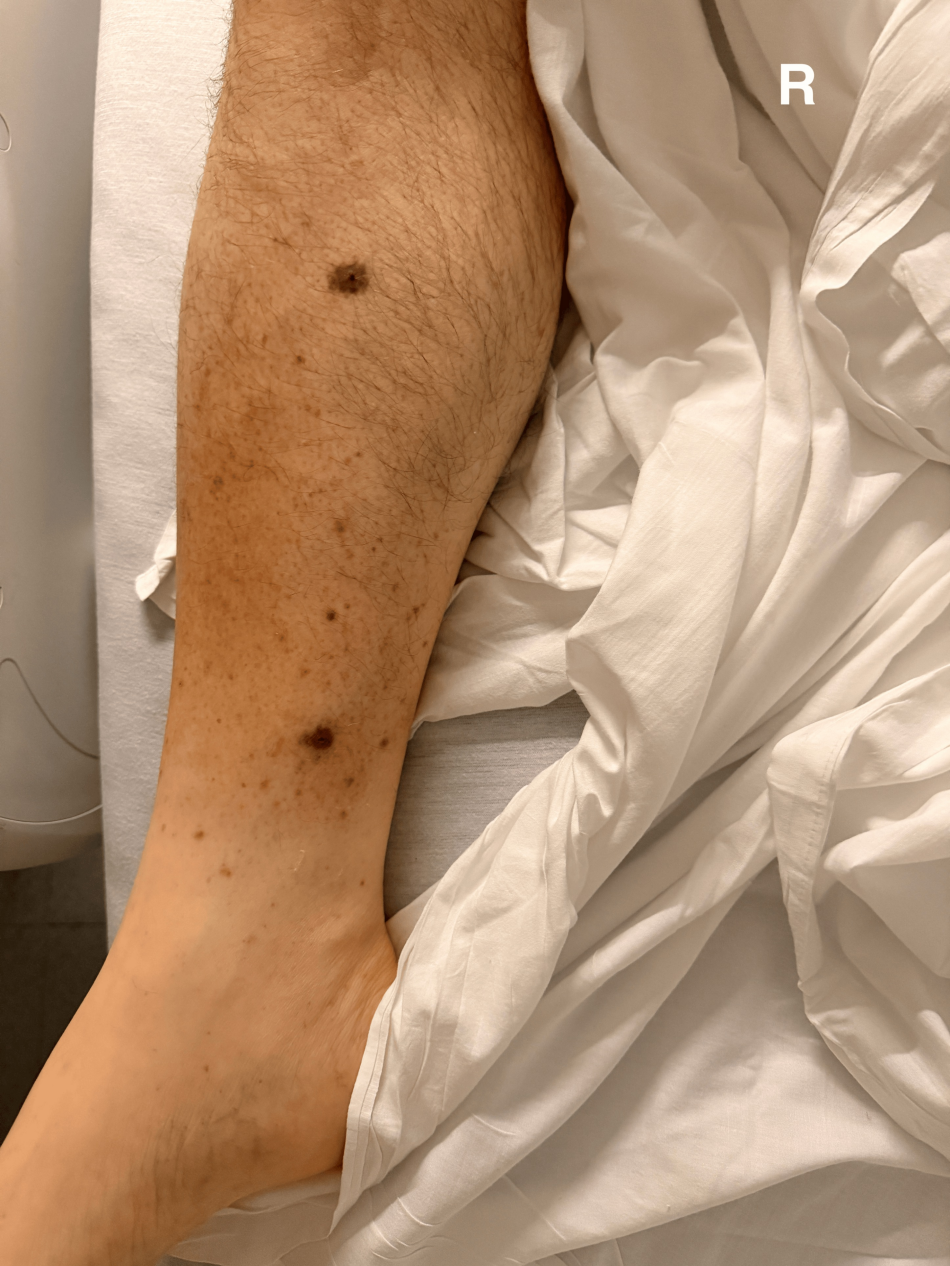
Physical examination revealed a maculopapular rash over the right lower extremity.

Physical examination demonstrated marked pallor, mild facial swelling, and a maculopapular rash over the lower extremities (Figures 1, 2).

Initial laboratory testing demonstrated profound cytopenias and hyperleukocytosis (Table 1).

**Table 1:** Initial laboratory testing demonstrated profound cytopenias and hyperleukocytosis. Abbreviations: WBC, white blood cell count; LDH, lactate dehydrogenase; AST, aspartate aminotransferase; ALT, alanine aminotransferase; PCR, polymerase chain reaction.

Parameter	Result	Reference Range	Interpretation
WBC	132.9 × 10⁹/L	4–11 × 10⁹/L	Marked leukocytosis
Hemoglobin	5.4 g/dL	13–17 g/dL	Severe anemia
Platelets	11 × 10⁹/L	150–400 × 10⁹/L	Severe thrombocytopenia
Lactate	6.5 mmol/L	<2.0 mmol/L	Hyperlactatemia
Uric acid	13.0 mg/dL	3.4–7.0 mg/dL	Hyperuricemia
Creatinine	2.1 mg/dL	0.6–1.3 mg/dL	Acute kidney injury
LDH	2291 U/L	135–225 U/L	Markedly elevated
AST	48 U/L	<40 U/L	Mild elevation
ALT	26 U/L	<40 U/L	Normal
COVID-19 PCR	Positive	Negative	Active infection

Peripheral blood smear performed at admission demonstrated circulating myeloblasts with Auer rods, consistent with AML. Approximately 36%-40% of blasts were reported. Confirmatory flow cytometry demonstrated a myeloid blast population expressing CD34, CD13, CD33, CD38, CD45, CD117, and HLA-DR, consistent with AML. Original flow cytometry plots were not available for retrieval from the laboratory archive at the time of manuscript preparation.

Bone marrow biopsy with cytogenetic and molecular profiling was deferred to the receiving tertiary oncology center following initial stabilization. These results were not available at the time of manuscript preparation.

No lymphadenopathy or organomegaly was noted. Neurologically, he was alert but fatigued, oriented, and without focal deficits.

**Figure 3:**
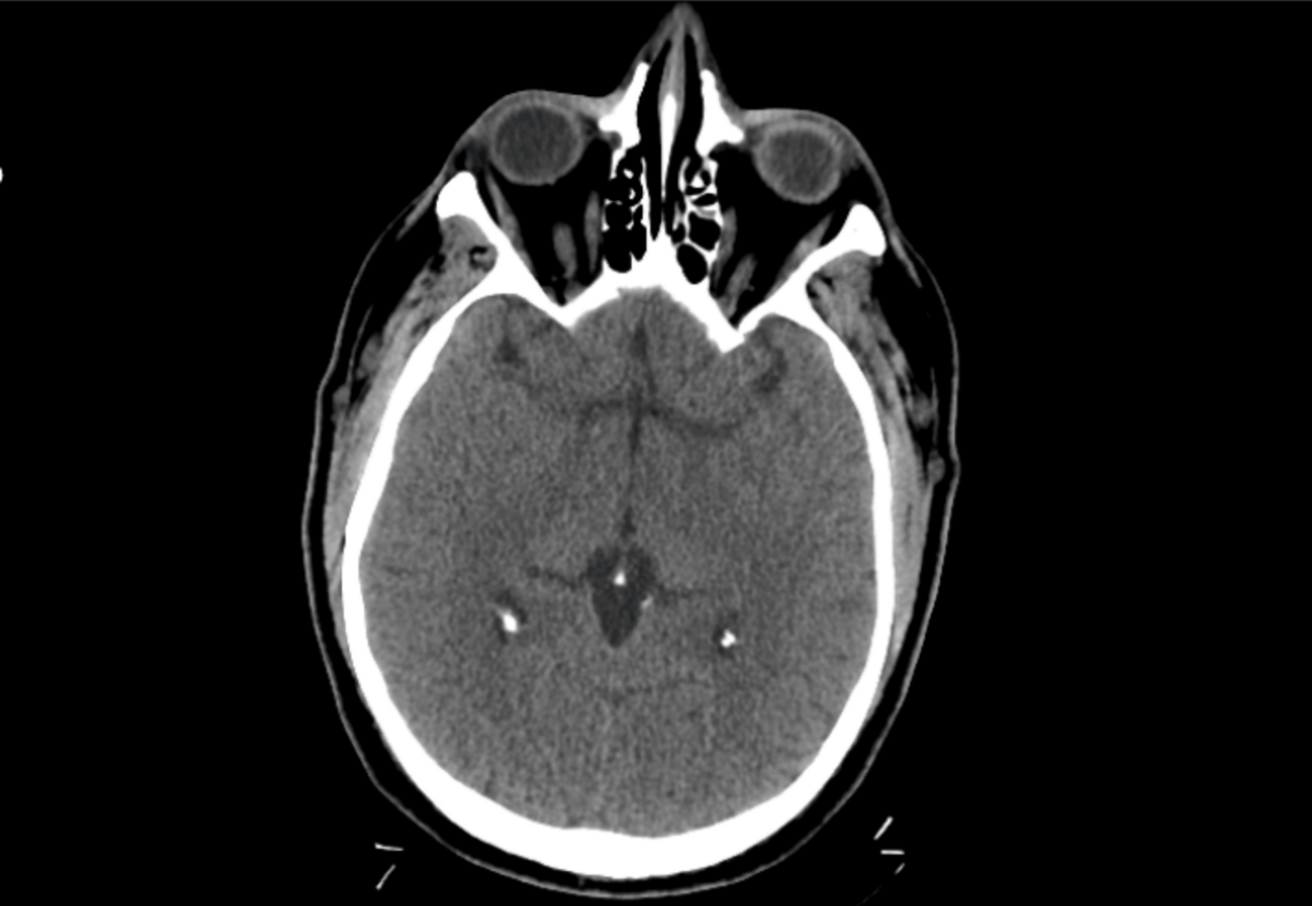
Non-contrast CT of the head showed no intracranial hemorrhage, infarct, or mass.

Non-contrast CT of the head showed no acute intracranial pathology (Figure 3). Further neurologic evaluation, including MRI brain and electroencephalography (EEG), was not performed prior to transfer. Lumbar puncture was deferred due to severe thrombocytopenia (platelet count 11 × 10⁹/L) and associated bleeding risk.

**Figure 4:**
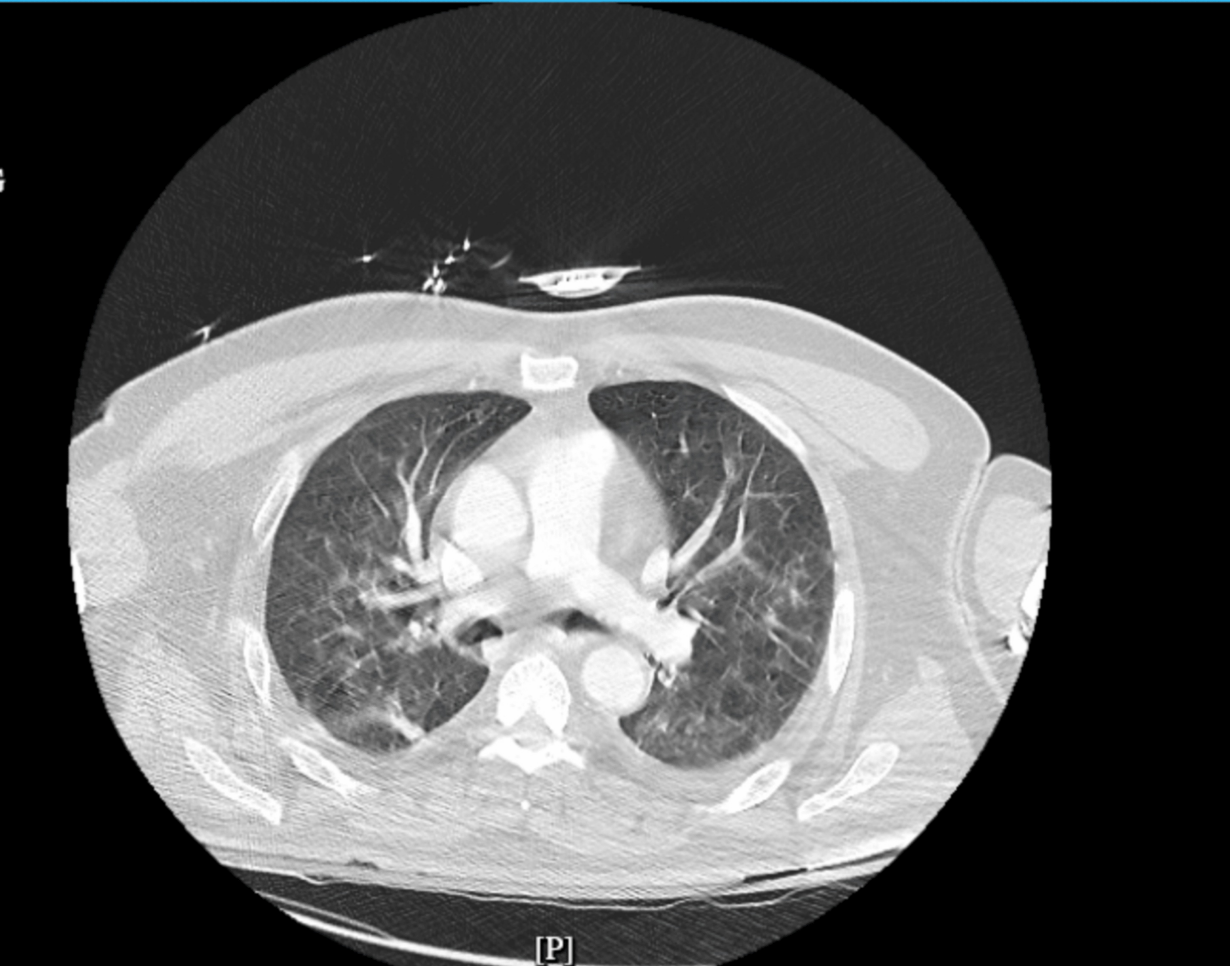
CT angiography of the chest was nondiagnostic for pulmonary embolism but showed small pleural effusions and mild bilateral ground-glass opacities consistent with COVID-19 pneumonitis.

CT angiography of the chest revealed mild bilateral ground-glass opacities and small pleural effusions compatible with COVID-19 pneumonitis (Figure 4).

**Figure 5:**
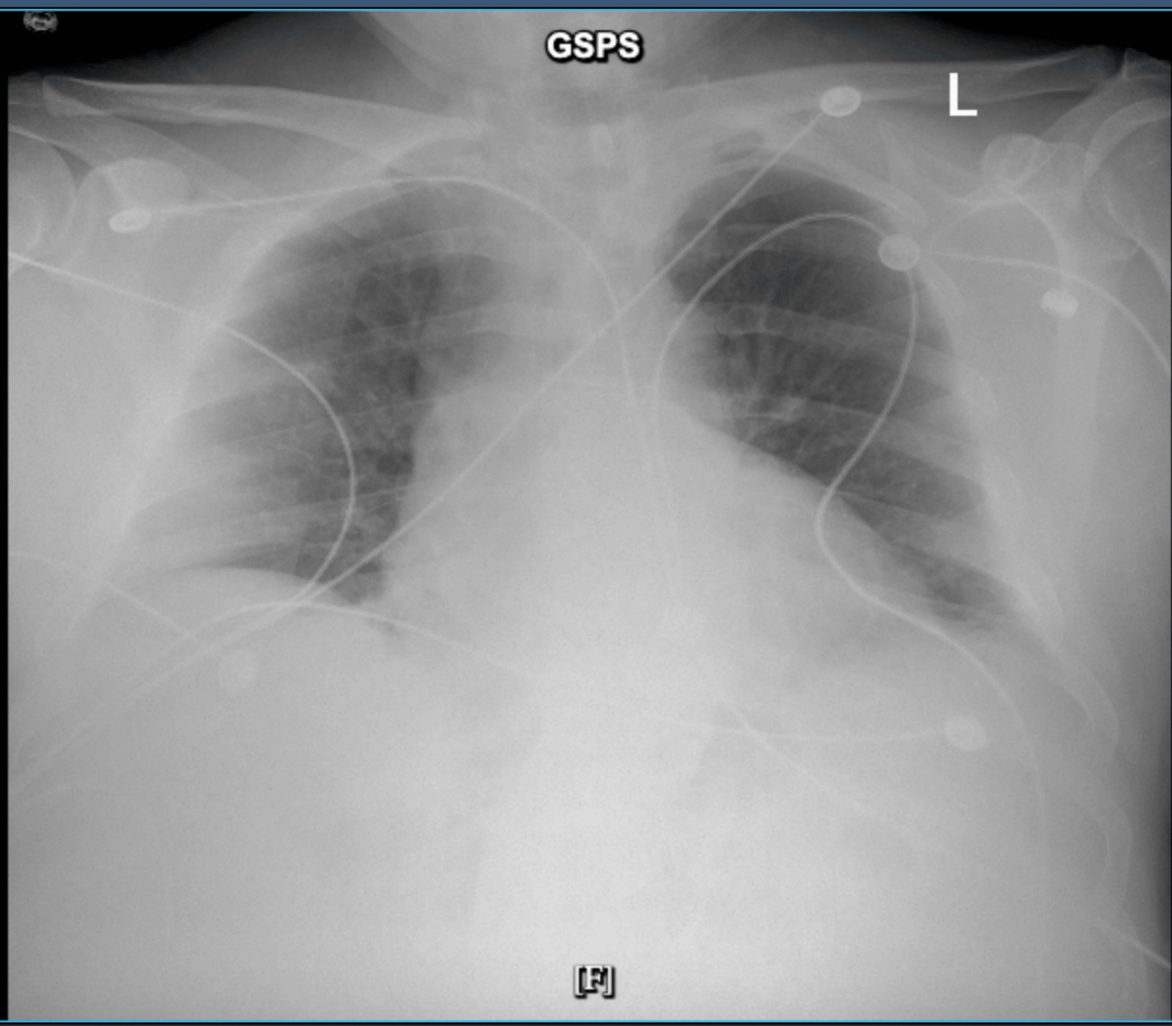
A chest radiograph demonstrated cardiomegaly and left basilar opacities. The image shows an enlarged cardiac silhouette and hazy opacities in the left lower lung zone, compatible with atelectasis or COVID-19-related pneumonitis.

Chest radiography demonstrated cardiomegaly with left basilar opacities (Figure 5).

Hospital course

The patient was admitted to the intensive care unit for close monitoring. Hydroxyurea (1 g every eight hours) was initiated for cytoreduction. Supportive management included intravenous fluids, allopurinol for tumor lysis prophylaxis, cefepime, doxycycline, remdesivir, and dexamethasone.

Transfusion support consisted of two units of packed red blood cells and multiple platelet transfusions, targeting a hemoglobin level greater than 7 g/dL and a platelet count greater than 20 × 10⁹/L.

Over the first 72 hours, the leukocyte count decreased from 132.9 × 10⁹/L to 32.5 × 10⁹/L. Hemoglobin improved to 7.1 g/dL, while platelet counts transiently increased to 45 × 10⁹/L following transfusion, although thrombocytopenia persisted. Serum creatinine normalized from 2.1 mg/dL to 1.0 mg/dL, and metabolic parameters improved.

The patient’s neurologic status stabilized without recurrent seizures. After clinical improvement and initial cytoreduction, he was transferred to a tertiary oncology center for definitive induction chemotherapy.

## Discussion

Hyperleukocytosis in AML represents a critical hematologic emergency associated with early mortality. Increased leukocyte burden results in impaired microcirculatory flow, endothelial damage, and tissue hypoxia, particularly affecting the central nervous system and lungs [1,3]. Although leukostasis-related microvascular obstruction is a causal mechanism for seizures, alternative contributors, including severe anemia, hyperlactatemia, and uremia, may have also played a role.

Management of hyperleukocytic AML focuses on rapid cytoreduction and prevention of complications. Hydroxyurea is widely accepted as first-line therapy due to its rapid onset of action and favorable safety profile [2].

Leukapheresis has historically been used and remains a consideration in patients with symptomatic leukostasis, particularly with central nervous system involvement. In this case, it was not pursued due to rapid cytoreductive response to hydroxyurea, absence of focal neurologic deficits, and concern for procedural risks in the setting of severe thrombocytopenia. Additionally, recent analyses have not demonstrated a consistent survival benefit [2].

Central nervous system involvement in acute leukemias further contributes to neurologic complications and poor outcomes [6]. Tumor lysis prophylaxis is essential in patients with elevated uric acid, lactate dehydrogenase, or renal dysfunction [7].

Transfusion strategies must balance correction of cytopenias with the risk of increased blood viscosity. Restrictive thresholds such as maintaining hemoglobin above 7 g/dL and platelets above 20 × 10⁹/L are recommended in hyperleukocytic AML [8].

Concurrent COVID-19 infection may have contributed to immune dysregulation that unmasked previously undiagnosed leukemia. SARS-CoV-2 infection is associated with cytokine overproduction and inflammatory signaling pathways that may promote proliferation of myeloid precursors [9].

Several studies during the COVID-19 era have described outcomes and management challenges in patients with acute leukemia and concurrent viral infections [9]. Additionally, the absence of bone marrow biopsy, cytogenetic, and molecular profiling data limits full classification of AML according to current WHO criteria and represents a limitation of this report [10].

## Conclusions

This case significantly highlights the importance of maintaining a high index of suspicion for hematologic emergencies in patients presenting with unexplained cytopenias, hyperleukocytosis, or neurologic symptoms, even in the setting of concurrent viral infection. AML with hyperleukocytosis can rapidly progress to life-threatening complications, including leukostasis, tumor lysis syndrome, and multiorgan dysfunction. SARS-CoV-2 infection may act as a physiologic stressor that contributes to the clinical manifestation of previously undiagnosed leukemia; however, causality cannot be established based on a single case.

Prompt recognition, early cytoreduction, aggressive tumor lysis prophylaxis, and multidisciplinary critical care management are essential to reduce early mortality. Early transfer to a specialized oncology center for definitive induction therapy is crucial for optimal outcomes. This case also underscores the need for further research into the interaction between viral infections and hematologic malignancies, particularly in the context of emerging infectious diseases.
